# Some Problems of To-Day

**Published:** 1918-05-25

**Authors:** 


### SOME PROBLEMS OF TO-DAY.

### Child-Labour and Health.

By A SOCIAL WORKER.

The strength of a chain can be only that of its weakest link. One weak link in the chain of care for the nation's children from before birth to adult life has lately been found in the too frequent neglect of the ex-baby. Another is now seen to lurk in the permitted overstrain of a number of children, especially of boys, by long hours of work during the actual school years. It is important now to call attention to this danger because it is natural enough in war-time that people should ask whether intelligent and capable children are not best employed in "doing their bit" by replacing someone who can go off to definite national service. They will reason, too, that work is in itself an education, and especially so if boy or girl takes it up at an age when thorough practical teaching can be deliberately given and dutifully accepted.

### The Uses of Blind-alley Occupations.

Like all social questions this one is many-sided, and as soon as a conclusion appears quite obvious a contradiction may upset it. We may remember an instance of this in the case of the Act familiarly known as the Children's Drink Bill. It seemed so obviously undesirable to send a child to fetch beer from the public-house! But it has often been a serious dilemma that worse results followed if the messenger were father, who perhaps did not come back, or mother, who left the house unguarded, or older sister. In the case of employed children, it has seemed unquestionable that blind-alley occupations such as those of golf caddie and errand boy or van boy might create the loafer. But experience shows that if these jobs were entirely barred it might be very difficult to find employment for town boys for whom out-door and not sedentary life is medically ordered. To reduce them to idleness and mischief would create a new evil.

This point is one of the many important ones raised at the conference held last July at the Board of Education Offices on the working of the Choice of Employment Act of 1910. The Juvenile Employment Officer for Birmingham related that the result of an active propaganda against blind-alley employments by press articles and addresses to parents and children was such that the van and errand boy threatened to become extinct. The goods department of one of the railway companies found itself short by some 200 boys. As a consequence the company took measures to remove drawbacks. Satisfactory boys were to be passed on into men's work in due course, provision for messing, protective clothing, and efficient supervision was made. Some extremely dirty work in trades to which naturally the less satisfactory boys and girls tended to gravitate was similarly made less pernicious by improved arrangements, while the influence of actual prohibitions by the Home Office had called attention to removable dangers. French polishing, for instance, may involve dust which is hurtful to the chest, but ventilation and dust extraction may make the work safe for a normal adolescent. Elsewhere members of golf clubs have made plans for their caddies. Attention has been given also to such matters as weights lifted and carried by employed juveniles, sources often of later-developing mischief.

### Overwork by School-children.

What is needed now is that a similar public interest should be awakened with regard to the injury to health and development that is certain to result from overwork by school-children. The Employment of Children Act of 1903 gave power to local authorities to forbid the work of children—boys and girls under fourteen—in such places as public-houses, theatres of all kinds, billiard-rooms, kitchens of restaurants and hotels, barbers' shops. But these powers are by no means used everywhere. Nor, unfortunately, can it generally be said that if parents allow their children to take work under trying conditions they will not allow overmuch of it. A century ago it was the action of fathers and mothers and not of Government or employers that sent actual infants into cotton and silk mills. The need of Care Committee work will increase rather than lessen for some time to come, till at least a stronger sense of the solidarity of humanity unites legislator and parent, teacher and employer in a common knowledge of children's needs and rights and purpose in securing them. Perhaps we begin to see the dawn of such feeling in many directions. On the religious side, for instance, a little card entitled "A Neighbour's Prayer" has lately been circulated in a North-Eastern diocese in England. After all, the religious and the social worker and a great number of public officials exist chiefly to do what their fellow-citizens ought not to have left undone. It is certain, for instance, that knowledge of the facts is the first, and ought to be the last thing needed to remove at once such scandals as were shown up at this conference by examples given of work done by Manchester children, by Mr. Spurley Hey, Director of Education in that city. Thus a lather boy, eleven years of age, works 43 hours per week—on schooldays 1 hour before 9, 1½ hours at mid-day, 3 hours evening, 12 hours Saturday, 4 on Sunday. Milk-boy, aged eleven, 48 hours, of which 12 on Saturday and 6 on Sunday. And a child of nine, 36¾ hours as greengrocer's boy. In none of these and other instances given was the child learning any craft in all these hours of toil, and we can hardly suppose that schooling could make much impression, while the effect on health and growth cannot be anything but harmful. Two serious faults in our present system are, first, that the child passes through the hands of too many separate authorities and caretakers. Secondly, that a good deal of our legislation is only permissive and needs to be made compulsory and therefore universal.

### Relief in the Education Bill.

The "increasing public desire to cherish our young people" with which Mr. Hey credits the nation should show itself in strong support to those clauses of the Education Bill which deal with children's employment and attention to needed amendments. As the Bill stands, no child is to work for hire till he is twelve. An amendment should provide that he should complete a school term in which his birthday occurs. It was a real father who called at 10.30 one morning to remove his son at the exact hour of his birth. Amendments are also needed to save the school child, the boy especially, from overwork on Saturdays and Sundays, otherwise fourteen hours may be inflicted on either day. Protection for young people whose education is to be continued is also needed. The medical secretary of a London Care Committee has lately shown that a number of school-leavers on his list, both boys and girls, have gone to spend ten to twelve hours daily in employment as messengers and so forth, or as trade learners. And in country places injury is certainly being done to schoolboys, who are employed for very long hours not in mere leisurely bird-scaring, but in heavy agricultural labour.

War-Time Conditions.

The Director of Education in Wiltshire voiced at the 1917 Conference the very general feeling that a time of difficulty in the country comes for girls between leaving school and entering domestic service when old enough to do so, and for boys at an age when they are no longer employable as boys. These conditions, of course, are temporarily altered by the war, and are less hurtful to physical than to mental and moral development. Continued education will do much to mend these and to facilitate the watching over and wise advising of young people going to work.

Mr. Bray, chairman of the London Advisory Committee, sees in the better food which better pay has secured a certain set-off against the overstrain to which multitudes of boys and girls have been subjected in war-work. There is no doubt that great efforts are wanted to promote the habit of regular and well-chosen meals among all young workers and in their homes, and also the habit of sufficient sleep, as one means of lessening the loss of health which will follow upon sudden relaxation and probably a term of unemployment.

